# High-Fat Diet and Alcohol Intake Promotes Inflammation and Impairs Skin Wound Healing in Wistar Rats

**DOI:** 10.1155/2018/4658583

**Published:** 2018-07-24

**Authors:** Daiane Figueiredo Rosa, Mariáurea Matias Sarandy, Rômulo Dias Novaes, Mariella Bontempo Freitas, Maria do Carmo Gouveia Pelúzio, Reggiani Vilela Gonçalves

**Affiliations:** ^1^Department of General Biology, Federal University of Viçosa, Viçosa, MG, Brazil; ^2^Department of Animal Biology, Federal University of Viçosa, Viçosa, MG, Brazil; ^3^Institute of Biomedical Sciences, Department of Structural Biology, Federal University of Alfenas, Alfenas, MG, Brazil; ^4^Department of Nutrition and Health, Federal University of Viçosa, Viçosa, MG, Brazil

## Abstract

The wound-healing process is complex and remains a challenging process under the influence of several factors, including eating habits. As improper diets may lead to disorders such as dyslipidemia, insulin resistance, and chronic inflammation, potentially affecting the tissue ability to heal, we decided to investigate the effect of a high-fat diet and alcohol intake on the inflammatory process and skin wound healing in Wistar rats. Male rats (*n* = 30) were individually housed in cages with food and water ad libitum (registration number 213/2014). After anesthesia, at day 40, three circular wounds (12 mm diameter) were made on the back of each animal, which were then randomly assorted into five treatment groups: C1 (control 1)—water via gavage and standard chow diet; C2 (control 2)—water (no gavage) and standard chow diet; AL (alcohol)—water (no gavage) and alcohol (40%) via gavage and standard chow diet; HF (high fat)—water (no gavage) and high-fat diet (50%); and HF + AL (alcohol/high fat)—water (no gavage), alcohol (40%) via gavage, and high-fat diet. Animals were treated for 61 days. Every seven days, the area and the rate of wound contraction were evaluated. Tissue samples were removed for histopathological analysis and biochemical analyses. Our results showed that wound contraction was not complete in the HF + AL rats. Two specific indices of wound-healing impairment (total cell number and levels of the inflammatory cytokine TGF-*β*) were increased in the HF + AL rats. We also observed decreased type I and III collagen fibers in the HF, AL, and HF + AL groups and increased oxidative stress markers in the same groups. We suggest that a high-fat diet combined with alcohol intake contributed to delayed skin wound healing through increase of the inflammatory phase and promoting oxidative stress, which may have led to morphological alterations and impaired matrix remodeling.

## 1. Introduction

Skin wound healing is an essential dynamic process, which includes basically three stages: inflammation, proliferation, and tissue remodeling. During the inflammatory stage, cell migration, cytokines, and growth factors play an important role as inflammatory mediators on vascular proliferation and therefore on tissue remodeling [1]. Chemical mediators are known to participate actively in wound healing during all three stages [2]. Among these mediating substances is the transforming growth factor beta (TGF-*β*), which has chemoattractant activity for macrophages, keratinocytes, and fibroblasts. TGF-*β* also stimulates the release of other growth factors and angiogenesis and inhibits proteolytic enzymes [3, 4].

During skin healing, fibroblasts, keratinocytes, and endothelial cells are recruited to synthesize the new tissue and enhance wound contraction [5]. At the beginning, the extracellular matrix is synthesized in order to repair the epidermal barrier [6]. Fibroblasts are the main cells involved with collagen III-rich granulation tissue, which is gradually replaced by the more resistant collagen I [7, 8].

Previous studies have demonstrated that, during skin wound healing, reactive oxygen species (ROS) are formed due to inflammation, especially at the beginning of the process [9, 10]. ROS production might be even more harmful when combined with unhealthy life habits and diseases such as diabetes [11]. Likewise, high-fat diets and alcohol intakes are associated with a number of malfunctions, as increased inflammatory phase, dyslipidemia, insulin resistance, and stroke, among others [12–14]. ROS formation usually inhibits the antioxidative defense system [15] and can damage cell structures as lipids, proteins, and DNA, altering the immune response and decreasing mediators' release [2]. The excessive ROS generation can lead to chronic inflammation, degeneration, and cell death, compromising the wound-healing process. In chronic inflammation, predominance of collagen III, glycosaminoglycans, and proteoglycans occurs in detriment to the deposition of collagen I, leading to the formation of a fragile scar, less resistant to traction [16].

Besides, other factors such as vascular alterations are also associated with high-fat diets and alcohol consumption, potentially affecting cell metabolism and tissue healing [17, 18]. Experimental models using the combined effects of these two dietary factors in preclinical studies are particularly useful for their potential applicability on clinical models [19, 20].

Considering that a high-fat diet and alcohol intake interfere on cellular metabolic pathways, we tested the hypothesis that the inflammation process would increase and wound healing would be impaired when these two dietary factors are combined. Therefore, the aim of this study was to evaluate the effects of a high-fat diet and alcohol intake on skin wound healing mainly in the inflammatory phase.

## 2. Methods

### 2.1. Animals

Healthy ten-week-old male Wistar rats (*Rattus norvegicus*) weighing an average of 320 g were randomly distributed in individual cages cleaned daily, at a constant temperature (22 ± 1°C) and a 12 : 12 h light : dark photoperiod. All procedures and protocols were approved by the Institutional Animal Care and Use Committee (CEUA/UFV—213/2014).

### 2.2. Experimental Design

Thirty rats were randomly distributed in 5 groups (*n* = 6 each): C1 (control 1)—water via gavage and standard chow diet; C2 (control 2)—water (no gavage) and standard chow diet; AL (alcohol)—water (no gavage) and alcohol (40%) via gavage and standard chow diet; HF (high-fat)—water (no gavage) and high-fat diet (50%); and HF + AL (alcohol/high-fat)—water (no gavage), alcohol (40%) via gavage, and high-fat diet. All animals were treated for 61 days and had water and food ad libitum. Rats from the AL and HF + AL groups received absolute alcohol (Dinâmica®) diluted in water to 40% [21] via gavage once a day. Rats from the HF and HF + AL groups were offered a prepared diet consisting of ham paste, potato fries, bacon, chocolate powder, cookies, milk powder, and commercial chow (containing 111 g of each one of the ingredients). These values were calculated for each 1000 g of diet and represent a total of 50% of fat, 20% of protein, and 30% of carbohydrates [19]. All diets were prepared in an aseptic environment using an industrial mixer for homogenizing. Animals were weighted every 7 days during the experiment. Food intake was calculated as the difference between food offered and the remaining leftovers after 24 h (g).

### 2.3. Skin Wounds

**Figure 1:**
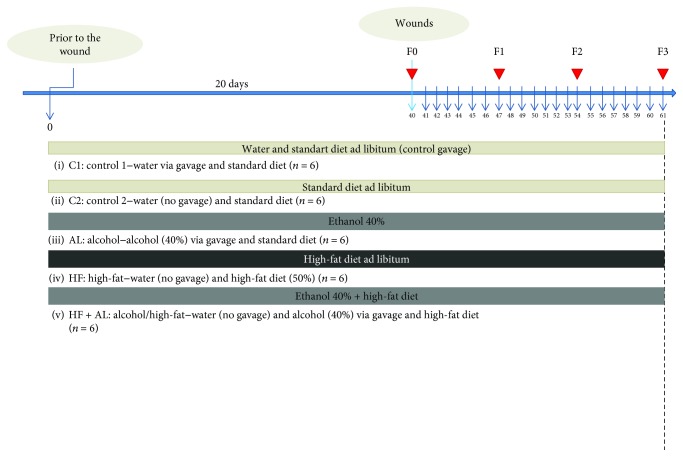
Flowchart of the experimental design with the distribution of the animals in the groups, treatment time (chronological line, blue), and wound performance and fragment collection (arrowhead in red). Thirty Wistar rats were randomized into five experimental groups: C1 = control 1—water via gavage and standard chow diet; C2 = control 2—water (no gavage) and standard chow diet; AL = alcohol—water (no gavage), alcohol (40%) via gavage and standard chow diet; HF = high-fat—water (no gavage) and high-fat diet (50%); HF + AL = alcohol/high-fat—water (no gavage), alcohol (40%) via gavage and high-fat diet. F0 = intact tissue; F1, F2, and F3 = scar tissue after 7, 14, and 21 days, respectively.

After 40 days of treatment, the animals were anesthetized with an intramuscular injection of ketamine (50 mg/kg) and xylazine (20 mg/kg) and three circular skin wounds of 12 mm diameter were created by surgical incision to expose the dorsal fascia using scalpel blade number 15. The wound area was previously marked with the use of crystal violet and measured with the use of an analog caliper (Mitutoyo Ltd.®, São Paulo, Brazil). The rats had their backs shaved, and the area was defatted with ethyl ether (Merck®, Rio de Janeiro, Brazil). Subsequently, we used 70% ethanol and 10% povidone-iodine for local asepsis [10]. In all groups, the wounds were cleaned with saline 0.9% for 21 days. Tissue samples (*n* = 6 in each day) were obtained from different wounds at days 7, 14, and 21 for histological, biochemical, and cytokine expression analysis. A tissue sample was removed on the first day of the experiment (F0) and stored for analysis of the uninjured tissue. A sample of the first (F1), second (F2), and third (F3) wounds of each animal was removed on the seventh, 14th, and 21st days, respectively, and each fragment removed was divided into three parts, to realize the proposed analyses.  Figure 1 shows the experimental design used to evaluate the time-dependent effects of alcohol and high-fat diet intake on wound healing in rats.

At the end of the experiment, all animals were euthanized by cardiac puncture under anesthesia with intraperitoneal ibuprofen (10 mg/kg). Wound area was calculated using computerized planimetry scanned images (DSC-W610®, Sony, Tokyo, Japan) and Image-Pro Plus 4.5® (Media Cybernetics, Silver Spring, MD, USA), previously calibrated. The rate of wound contraction was calculated using the following ratio: {[initial wound area(A0) − area at the day of measure(Ai)]/initial wound area(A0)} × 100. The third wound was selected for analysis of the healing process, for having fragments removed only in the last day of the experiment.

### 2.4. Histological Analysis

Tissue fragments were removed for histological analysis and fixed in Karnovsky solution, dehydrated in ethanol, cleared in xylene, and embedded in paraffin. Sections (4 *μ*m thick) were obtained in a rotary microtome (Leica Multicut 2045®, Reichert-Jung, Jena, Germany), using 1 in 20 sections to avoid repetition of the analysis of the same histological area. The sections were stained with hematoxylin and eosin (HE) for analysis of fibroblasts and blood vessels, and Sirius Red (Sirius F3B red, Mobay Chemical Co., Union, NJ, USA) for analysis of type I and III collagen fibers under microscope polarization (Sigma-Aldrich, St. Louis, Missouri, USA) [22]. To highlight the elastic fibers, sections of tissue were stained by the Verhoeff method [23]. Staining for hemosiderin was performed by iron marking by Perls stain [24] and 1% neutral red. Images were captured by a camera (Olympus DP73) attached to a bright field microscope BX53® (Olympus, Tokyo, Japan) and analyzed with cellSens Dimensions and Image-Pro Plus® software. Using a 20x objective lens, 10 histological fields were randomly selected in each skin section and an area of 1.53 × 10^6^ *μ*m^2^ from the tissue was analyzed. The analysis consisted in counting all structures of interest in a pattern area of 153 × 10^3^ *μ*m^2^. For analysis of mast cells, scar tissue sections were stained with toluidine blue [25]. Using a 40x objective lens, 10 histological fields were analyzed with a total area of 1.96 mm^2^ under a light microscope CX40® (Olympus, Tokyo, Japan). Each histological section showed an area of 0.196 mm^2^. This area was calculated from the number of the field = 20/40, and then the formula *A* = *πr*^2^ was used.

### 2.5. Expression of Inflammatory Cytokine TGF-*β*

Scar tissue samples collected on days 7 and 14 were frozen at −80°C and homogenized in PBS buffer (pH 7.4) containing Tween (0.05%) and centrifuged at 3500*g* for 30 min. TGF-*β* levels in the supernatant were analyzed using ELISA immunoassay kits (Boster Biological Technology Ltd., China), following the manufacturer's protocol. High-affinity polystyrene plates (Corning, New York, USA) were coated with 100 *μ*L/well of specific monoclonal antibodies (capture antibody) diluted in 0.1 M carbonate-bicarbonate buffer (pH 9.6) for 12 h at 4°C. Plates were then blocked with PBS solution and 10% inactivated fetal bovine serum (Sigma-Aldrich) was added for 1 h at room temperature. In each well, recombinant compounds were added in duplicate (standard curve) followed by the samples of scar tissue homogenate. The plates were incubated at room temperature for 2 h and then washed five times with PBS-Tween and added to a specific secondary antibody for each component of interest conjugated to biotin (detection antibody) associated with the avidin-peroxidase. The reaction was developed with tetramethylbenzidine (TMB) and blocked with 2 M sulfuric acid after 20 min. The reading was performed in a microplate reader at 450 nm (Power Wave X BioTek Instruments Inc., Winooski, VT, USA).

### 2.6. Oxidative Stress

Tissue fragments were collected from each wound, quickly frozen in liquid nitrogen (−196°C), and stored at −80°C. Samples were homogenized in phosphate buffer and centrifuged at 5°C at 3500*g* for 10 min, and the supernatant was used for analysis of thiobarbituric reactive substances (TBARS) and protein carbonyls (PCN) as well as superoxide dismutase (SOD), catalase (CAT), and glutathione-S-transferase (GST) activities. TBARS was performed according to the protocol described by Halliwell and Gutteridge [26]. Carbonyl protein levels were determined by the method adapted from Jana et al. [27]. The supernatant was incubated for 15 min with 500 *μ*L of 2,4-dinitrophenylhydrazine (10 mM DNFH prepared in 2 M HCl). After incubation, proteins were precipitated with 500 *μ*L of 10% trichloroacetic acid, centrifuged for 10 min at 3500*g*. The precipitate was resuspended in 1 mL of 6% SDS solution for 10 min and centrifuged at 5000*g*. Supernatant was collected for reading. Protein carbonyls were quantified spectrophotometrically by reading at 700 nm. SOD activity was performed using an ELISA reader set at 570 nm, based on the ability of this enzyme to catalyze the reaction of superoxide (O^2−^) in hydrogen peroxide, thereby decreasing the rate of autooxidation of pyrogallol [28]. CAT activity was assessed using the Aebi method [29], by measuring the rate of decomposition of hydrogen peroxide (H_2_O_2_). GST was measured through the formation of glutathione-2,4-dinitrobenzene torque and estimated by the change in absorbance at 340 nm for 60 s. The formation of the conjugate occurs spontaneously on the substrate 1-chloro-2,4-dinitrobenzene (CDNB) in nonenzymatic reaction and is accelerated by the activity of GST. One unit (U) of GST is the amount of enzyme which forms 1 mol of glutathione-2,4-dinitrobenzene conjugate per min. The molar extinction coefficient of CDNB_340_ = 9.6 mM^−1^ cm^−1^ was used for calculation [30], and the results were expressed as *μ*mol min^−1^ g^−1^. The biochemical data were normalized to total protein levels in the supernatant [31].

### 2.7. Blood Marker Tests

Blood was collected during euthanasia, after 12 h of fasting, through cardiac puncture using a heparinized syringe. Blood samples were then centrifuged at 1000*g* for 10 min, and the serum was reserved for the levels of triglyceride analyses, glucose, alanine aminotransferase (ALT), and aspartate aminotransferase (AST). Diagnostic kits used were purchased from BioClin® (Belo Horizonte, MG, Brazil). Serum was analyzed at the Clinical Analysis Laboratory, Department of Nutrition and Health of the Federal University of Viçosa, using a clinical analyzer BS-200 Mindray® (China).

### 2.8. Statistical Analysis

The results were expressed as the mean ± standard deviation (SD). Normal distribution of data was assessed using the D'Agostino-Pearson test. The sample size calculation was based on nonstandard deviation, significance level 5%, and test power of 95% assuming that variables are quantitative and unstable. To define the sample size, we used the WinPepi program, with its significance of 5% and a certainty of 10% [32]. Parametric data were submitted to one-way analysis of variance (one-way ANOVA) followed by Student-Newman-Keuls test for multiple comparisons. Nonparametric data were analyzed using the Kruskal-Wallis test. Significance was set at *p* ≤ 0.05. All data were analyzed using GraphPad Prism 5 (Prism Software, Irvine, CA, USA).

## 3. Results

### 3.1. Wound Area and Histopathological and Inflammatory Results

**Figure 2:**
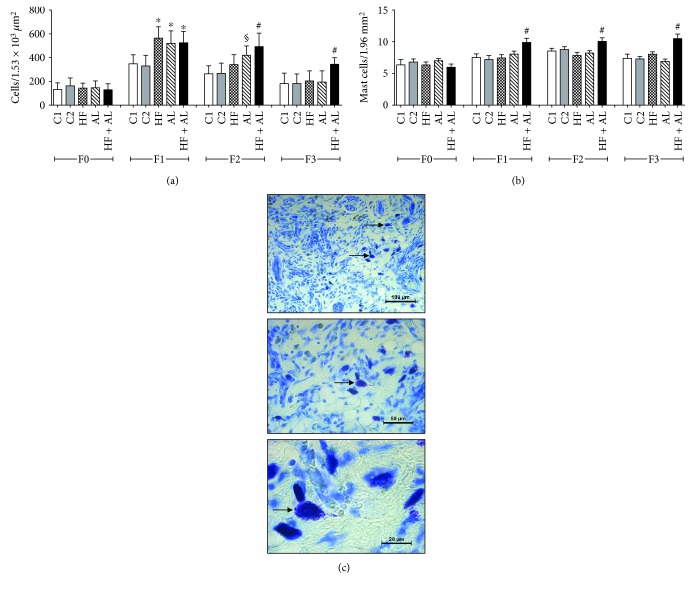
Effects of alcohol and high-fat diet on the total amount of cells (a), number of mastocytes (b), and mast cells in the scar tissue of HF + AL (c) in skin wounds of Wistar rats. C1 = control 1—water via gavage and standard chow diet; C2 = control 2—water (no gavage) and standard chow diet; AL = alcohol—water (no gavage), alcohol (40%) via gavage and standard chow diet; HF = high-fat—water (no gavage) and high-fat diet (50%); HF + AL = alcohol/high-fat—water (no gavage), alcohol (40%) via gavage and high-fat diet. F0 = intact tissue; F1, F2, and F3 = scar tissue after 7, 14, and 21 days, respectively. ∗ indicates statistical differences versus C1 and C2; § indicates statistical difference versus C1, C2, HF, and HF + AL; # indicates statistical difference versus C1, C2, AL, and HF. Arrows show the distribution of mast cells in the cicatricial tissue of HF + AL mice during F3 periods (c). Toluidine blue, bar: 100 *μ*m (10x), 50 *μ*m (20x), and 20 *μ*m (100x).

Rats from the HF, AL, and HF + AL groups had an increased wound area on days 7, 14, and 21 when compared to the control groups (Table 1). There were no significant differences in wound contraction rate in any treatment groups. On day 7 (F1), the total number of cells was higher in HF, AL, and HF + AL when compared to C1 and C2. On day 14 (F2), the AL and HF + AL groups showed a higher number of cells when compared to the other groups. On day 21 (F3), the number of cells was higher in HF + AL when compared to the others (Figure 2(a)). Regarding the number of mast cells on days 7, 14, and 21, this number was a higher in the HF + AL group when compared to the other groups (Figure 2(b)). These results can be confirmed by the analysis of Figure 2(c).

**Figure 3:**
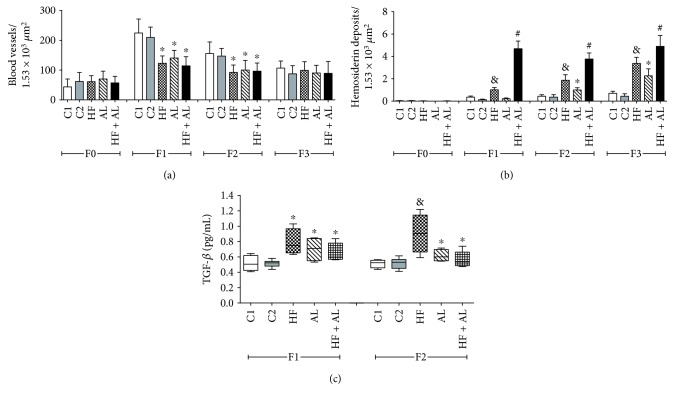
Effects of alcohol and high-fat diet on the total amount of blood vessels (a) and hemosiderin deposits (b) and TGF-*β* levels (c) in skin wounds from Wistar rats. C1 = control 1—water via gavage and standard chow diet; C2 = control 2—water (no gavage) and standard chow diet; AL = alcohol—water (no gavage), alcohol (40%) via gavage and standard chow diet; HF = high-fat—water (no gavage) and high-fat diet (50%); HF + AL = alcohol/high-fat—water (no gavage), alcohol (40%) via gavage and high-fat diet. F0 = intact tissue; F1, F2, and F3 = scar tissue after 7, 14, and 21 days, respectively. ∗ indicates statistical differences versus C1 and C2; # indicates statistical difference versus C1, C2, AL, and HF; & indicates statistical differences versus AL and HF + AL.

**Figure 4:**
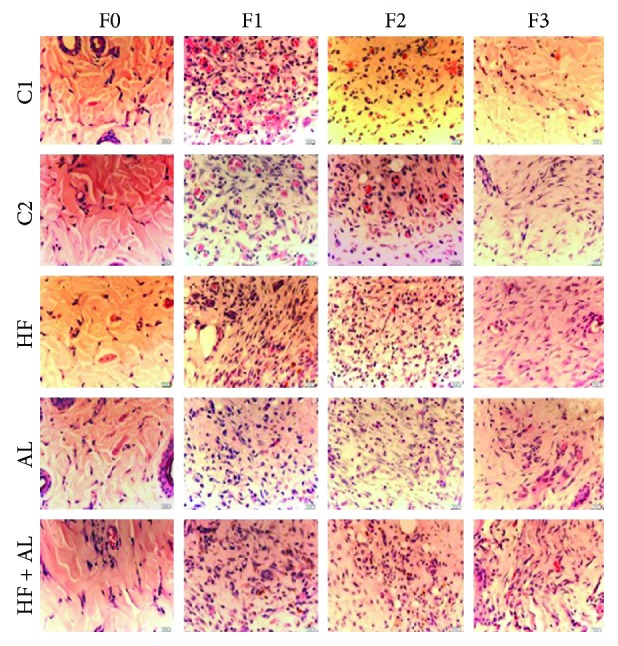
Photomicrographs showing blood vessel distribution on scar tissue in Wistar rats. C1 = control 1—water via gavage and standard chow diet; C2 = control 2—water (no gavage) and standard chow diet; AL = alcohol—water (no gavage), alcohol (40%) via gavage and standard chow diet; HF = high-fat—water (no gavage) and high-fat diet (50%); HF + AL = alcohol/high-fat—water (no gavage), alcohol (40%) via gavage and high-fat diet. F0 = intact tissue; F1, F2, and F3 = scar tissue after 7, 14, and 21 days, respectively. Bar: 20 *μ*m. HE staining.

On days 7 and 14, there were fewer blood vessels in the HF, AL, and HF + AL groups when compared to the controls (C1 and C2) (Figure 3(a)). The results described above corroborate those from Figure 4, showing decreased number of blood vessels and increased number of cells in tissues from animals treated with alcohol and high-fat diet. The amount of hemosiderin on day 7 (F1) was increased in HF and HF + AL when compared to the other groups. On day 14 (F2), the amount of iron deposits in HF, AL, and HF + AL was increased when compared to the controls. Besides, HF + AL had even higher iron deposits than the HF and AL groups. Similar results to those found on day 14 were observed on day 21 (Figure 3(b)).

We also observed increased values of TGF-*β* on day 7 in the HF, AL, and HF + AL groups compared to controls. On day 14, TGF-*β* values found in HF, AL, and HF + AL rats were higher than those in the control groups, with a highlight for the HF group, which showed values even higher than all other groups (Figure 3(c)).

**Figure 5:**
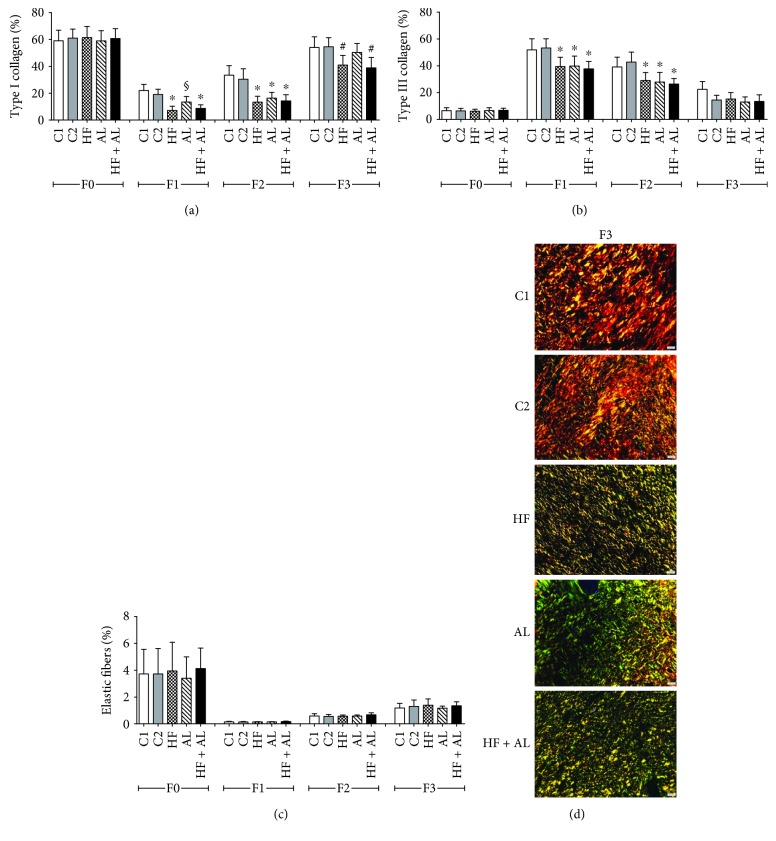
Effects of alcohol and high-fat diet on the total amount of type I collagen (a), type III collagen (b), and elastic fibers (c) in skin wounds of Wistar rats. Photomicrographs taken under polarized light demonstrating collagen distribution on scar tissue, showing red fibers (collagen I) and green fibers (collagen III) (d). C1 = control 1—water via gavage and standard chow diet; C2 = control 2—water (no gavage) and standard chow diet; AL = alcohol—water (no gavage), alcohol (40%) via gavage and standard chow diet; HF = high-fat—water (no gavage) and high-fat diet (50%); HF + AL = alcohol/high-fat—water (no gavage), alcohol (40%) via gavage and high-fat diet. F0 = intact tissue; F1, F2, and F3 = scar tissue after 7, 14, and 21 days, respectively. Bar: 20 *μ*m. Sirius Red staining. ∗ indicates statistical differences versus C1 and C2; # indicates statistical difference versus C1, C2, and AL; § indicates statistical difference versus C1, C2, HF, and HF + AL.

Regarding the effects of the high-fat diet and alcohol consumption on type I and type III collagen fibers and on elastic fiber production, on days 7 and 14, there was a decrease in deposition of type I collagen fibers in HF, AL, and HF + AL when compared to controls (Figure 5(a)). Seven days later, the HF and HF + AL groups showed a decrease in fiber deposition compared to the other groups. Regarding type III collagen fibers, on days 7 and 14, groups HF, AL, and HF + AL showed decreased fiber deposition compared to controls (Figure 5(b)). The analysis of elastic fibers showed no differences among groups throughout the experiment (Figure 5(c)). The data described above are corroborated by the decreased number of red fibers (collagen I) found at the end of 21 days (F3) (Figure 5(d)).

### 3.2. Oxidative Stress Markers and Antioxidant Enzymes

**Figure 6:**
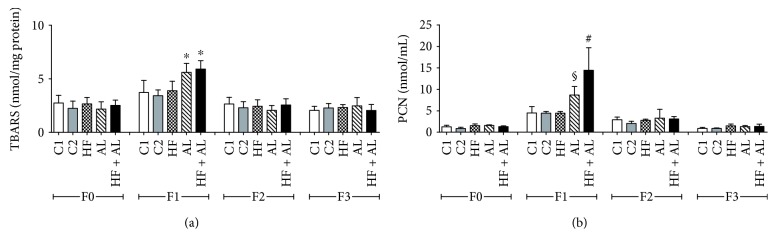
Effects of alcohol and high-fat diet on TBARS (a) and protein carbonyls (PCN) (b) in skin wounds from Wistar. C1 = control 1—water via gavage and standard chow diet; C2 = control 2—water (no gavage) and standard chow diet; AL = alcohol—water (no gavage), alcohol (40%) via gavage and standard chow diet; HF = high-fat—water (no gavage) and high-fat diet (50%); HF + AL = alcohol/high-fat—water (no gavage), alcohol (40%) via gavage and high-fat diet. F0 = intact tissue; F1, F2, and F3 = scar tissue after 7, 14, and 21 days, respectively. ∗ indicates statistical differences versus C1 and C2 and HF; # indicates statistical differences versus C1, C2, HF, and AL; § indicates statistical difference versus C1, C2, HF, and HF + AL.

Oxidative stress markers showed that, on day 7, AL and HF + AL had the highest values for TBARS and protein carbonyls (PCN) when compared to HF and both control groups (Figures 6(a) and 6(b)). On days 14 and 21, there was no difference among experimental groups.

**Figure 7:**
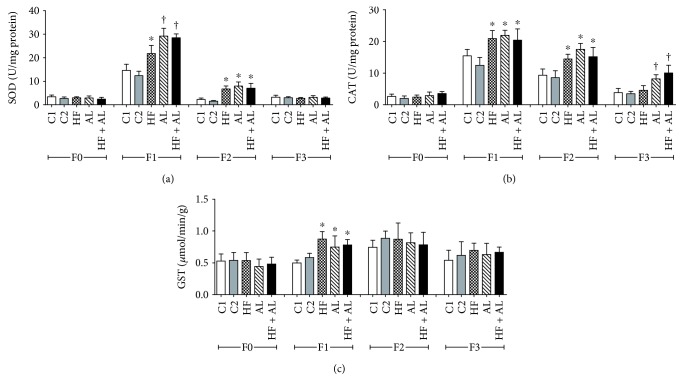
Effects of alcohol and high-fat diet on antioxidant enzymes SOD (a), CAT (b), and GST (c) in skin wounds from Wistar. C1 = control 1—water via gavage and standard chow diet; C2 = control 2—water (no gavage) and standard chow diet; AL = alcohol—water (no gavage), alcohol (40%) via gavage and standard chow diet; HF = high-fat—water (no gavage) and high-fat diet (50%); HF + AL = alcohol/high-fat—water (no gavage), alcohol (40%) via gavage and high-fat diet. F0 = intact tissue; F1, F2, and F3 = scar tissue after 7, 14, and 21 days, respectively. ∗ indicates statistical differences versus C1 and C2; † indicates statistical difference versus C1, C2, and HF.

The analysis of antioxidant enzymes showed that the HF, AL and HF + AL rats showed increased SOD and CAT activities on days 7 and 14, when compared to controls (Figures 7(a) and 7(b)). GST activity was also higher in the HF, AL, and HF + AL rats on day 7 as compared to controls (Figure 7(c)). An increase in stress markers may lead to chronic inflammation and cell death, contributing to tissuemorphological alterations and the formation of a fragile scar tissue.

### 3.3. Blood Markers

Blood tests showed an increase in triacylglycerol (TAG) concentrations in HF and HF + AL when compared to control groups (Table 2). The HF + AL rats showed even higher blood TAG when compared to those receiving high-fat diets alone. Blood glucose, AST, and ALT levels were higher in HF, AL, and HF + AL when compared to controls (Table 2).

## 4. Discussion

Our results suggest that a high-fat diet and alcohol intakes contributed to alterations in the connective tissue, inducing biochemical alterations that prevented proper tissue reconstruction within 21 days, and therefore impairing skin contraction and wound healing. This is the first study to report impaired wound healing in rats fed a combination of high-fat diet and alcohol intake, two items associated with bad eating habits. An unbalance on cell proliferation and migration, with increased chemical mediator release due to inflammatory processes, has been demonstrated in skin-healing models submitted to high-fat diet and alcohol [18, 19, 33]. We believed, likewise, an inflammatory condition was also observed here, since a sharp increase in cellularity was observed, especially during remodeling (21 days). These findings corroborate the increased number of mast cells in rats consuming both alcohol and a high-fat diet. Usually, the remodeling phase is characterized by decreased cell number due to intense tissue apoptosis, allowing the tissue to develop intact skin characteristics [34, 35]. As observed here, increased cell number was also shown in the skin from animals fed a diet rich in trans fats [36, 37] and in the stomach from rats that received high doses of alcohol [38], showing that the consumption of these items can lead to inflammatory infiltrate tissue and delay the healing process.

Tissue vascularity is a crucial factor for the healing process since it provides oxygen and nutrients for cells' metabolic activity [7, 39, 40]. Our results showed that rats receiving high-fat diet and alcohol had a lower vascularization and therefore a delay in wound closure. These results are similar to those found in mice receiving alcohol, in which a decrease in skin vascularization was observed [41]. Costa et al. [42] also reported decreased vascular endothelial growth factor (VEGF), which reduces formation of new blood vessels in diabetic animals receiving alcohol at 5%. In addition, high-calorie-diet administration in mice also seems to cause a decrease in VEGF expression, reducing tissue vascularization in wound healing [42, 43]. Interestingly, our results also showed an iron accumulation, forming hemosiderin deposits in the skin from animals that received the high-fat diet and alcohol simultaneously. One possible explanation for this finding would be a liver overload caused by alcohol and fat intake, impairing the biotransformation of bilirubin and interfering on its excretion through urine [44, 45]. This finding also corroborate important alterations found in the extracellular matrix in the groups that received alcohol and high-fat diet, since these components may occupy regions of the tissue that should be filled by collagen, leading to an inefficient deposition of fibers and consequently the formation of a thin, fragile scar.

Higher blood AST and ALT found in our study also seems to support a hypothesis of an overall malfunction of the body caused by excessive free-radical generation, delaying the healing process [43, 46]. The newly formed granulation tissue, along with cells and blood vessels, presents fibrillary components that support cell migration and act as a scaffold for tissue maturation, providing the new skin with resistance and strength [46–48]. Our results showed that the administration of a combined high-fat diet and alcohol delayed type I and type III collagen deposition, impairing cell migration and fiber synthesis and remodeling. A reduction in overall levels of collagen and hydroxyproline expression associated with increased metalloproteinase has been described in animals exposed to similar conditions [8, 34, 49]. Other studies also suggest that in addition to the decrease in number, a reduction in density and no alignment of the fibers also occur in response to a high-fat diet consumption, compromising biomechanical properties and the regeneration of connective tissue [50, 51]. These alterations are usually associated with the formation of a more fragile scar that is less resistant to shear stress [52]. *In vitro* studies have also shown that exposure to ethanol causes a reduction in cellular proliferation and lysyl oxidase activity, reducing hyaluronic acid content and hence the amount of collagen [48, 53, 54]. Changes in cell activities and matrix components are usually associated with alteration in growth factor synthesis. TGF-*β* is an important tissue marker that can be produced by various cells in response to an injury and is typically produced for short periods and in limited amounts, since its action takes place in distinctive phases of the process [55, 56]. Our results showed large amounts of TGF-*β* produced by groups receiving a high-fat diet and alcohol, contradicting the above statement that its production would be short and limited. Excessive production of TGF-*β* may lead to disorganized deposition of collagen fibers, usually observed during inflammation. This process may lead to an incorrect deposition of the extracellular matrix, keloid formation, and hypertrophic scars [57]. Similar results were found by Otranto et al. [58], who observed high levels of TGF-*β* in animals that received a high-fat diet for 14 days.

The findings we describe here confirm previous reports that skin trauma increases oxidative stress in the scar tissue [59, 60]. We found an increase in oxidative stress markers and antioxidant enzymes in the skin tissue on day 7 when rats were fed both a high-fat diet and alcohol, compared to controls. Excess free radicals are usually associated with an increased inflammatory phase, in a process known as respiratory burst, where macrophages produce free radicals and release into the tissue. Free radicals promote cellular oxidative stress, damaging membranes, proteins, and genetic material [33, 61]. In our study, the excess production of reactive species of thiobarbituric acid (malondialdehyde) and protein carbonyls showed a negative effect of combining a high-fat diet and alcohol on skin repair. Increases in these two oxidative stress markers indicate possible membrane and protein cell lesions, possibly due to a continuous effect of a stressor. These indicative alterations may include an initial inflammation with reversible lesions and later necrosis or apoptosis [15, 46, 60]. Acetaldehyde oxidation and pH alterations to the epidermis stratum corneum were also reported as factors extending the inflammation process in scar tissue when ethanol consumption is involved [2]. Increased tissue oxidative stress was also observed in studies using fat diets [13, 62]. According to Nascimento and Monte-Alto-Costa [36], obese mice had higher lipid peroxidation compared to normal weight mice, which indicates cell membrane disorganization in the overweight group. This increase in tissue oxidative stress may also be associated with diabetes or other changes in insulin levels [58]. Usually, membrane lipid destruction happens earlier than protein destruction, though the combination of these two markers confirms tissue degeneration and cell death [58]. Regarding the activities of the antioxidant enzymes, our results showed that the high-fat diet and alcohol consumption increased SOD, CAT, and GST activities, suggesting that skin is going through oxidative stress, as the defense system was shown to be active. Typically, when markers of oxidative lesions such as MDA and CP are elevated, antioxidant defense systems are also activated to neutralize free radicals, resulting in decreased markers of oxidative stress in tissues. However, when the effect of an aggressive agent is markedly intense, the antioxidant systems might not be able to neutralize the harmful ROS production and the tissues might present morphological and functional alterations that delay the healing process.

## 5. Conclusion

This study suggests that a high-fat diet and alcohol intake induced systemic and local alterations, possibly contributing to an inflammation process which might have led to impaired wound healing in rats. The main alterations found were increased blood markers, hemosiderin deposit in tissues, increased cell number, and enhanced inflammatory factor release following a high-fat diet and alcohol intake. In addition, we also observed impairments in the synthesis of ECM constituents, as decreased collagen fibers, delaying wound closure. These results suggest that a high-fat diet associated with excessive alcohol consumption may increase inflammation and delay the skin-healing process, contributing to a chronic process that culminates in a fragile, less resistant scar tissue formation. However, other studies with different diet contents and alcohol percentages are necessary to confirm our findings and extrapolate them to humans, since improper eating habits are currently a global issue, marked by increased intake of foods that are energy-dense but nutrient-poor [63].

## Figures and Tables

**Table 1:** Area (mm^2^) and wound contraction rate (WCR) (%) in all experimental groups at day 0 and after 7, 14, and 21 days of treatment. Values are mean ± SD.

		C1	C2	HF	AL	HF + AL
Day 0	Area	158.5 ± 26.5	153.3 ± 11.6	164.5 ± 13.6	163.3 ± 12.5	160.38 ± 12.6
	WCR	00.00 ± 00.00	00.00 ± 00.00	00.00 ± 00.00	00.00 ± 00.00	00.00 ± 00.00
Day 7	Area	76.8 ± 17.6	102.8 ± 31.6	113.1 ± 13.9^∗^	105.6 ± 8.2^∗^	115.4 ± 14.9^∗^
	WCR	50.51 ± 12.6	29.06 ± 11.7	39.77 ± 21.53	37.73 ± 13.1	31.9 ± 17.4
Day 14	Area	16.8 ± 9.1	15.9 ± 9.8	36.2 ± 11.9^∗^	30.3 ± 15.4^∗^	38.0 ± 12.3^∗^
	WCR	88.95 ± 5.5	89.16 ± 6.05	88.34 ± 7.96	81.30 ± 9.1	84.70 ± 9.29
Day 21	Area	3.2 ± 4.5	2.8 ± 1.7	9.1 ± 2.1^∗^	8.18 ± 4.6^∗^	10.4 ± 3.4^∗^
	WCR	96.73 ± 2.7	98.00 ± 1.2	94.06 ± 5.35	94.96 ± 2.61	94.38 ± 5.35

C1: control, water via gavage and standard chow diet; C2: control, water (no gavage) and standard chow diet; HF: water (no gavage) and high-fat diet (50%); AL: alcohol (40%) via gavage and standard chow diet; HF + AL: alcohol (40%) via gavage and high-fat diet. ∗ indicates statistical differences versus C1 and C2.

**Table 2:** Effects of alcohol and high-fat diet on serum glucose, triacylglycerol, total cholesterol, HDL cholesterol, AST, and ALT concentrations in Wistar rats.

mg/dL	C1	C2	AL	HF	HF + AL
Glucose	141.6 ± 25.3	144.8 ± 17.1	163.5 ± 23.4^∗^	159 ± 13.1^∗^	163.1 ± 20.5^∗^
Triacylglycerol	25.8 ± 3.9	33.83 ± 6.25	35.7 ± 9.7	37.6 ± 8.2^∗^	41.5 ± 11.2^∗^^#^
Total cholesterol	71.5 ± 12.1	60.1 ± 9.5	78.6 ± 10.6	60.3 ± 12.4	57.1 ± 20.5
HDL cholesterol	25.5 ± 2.9	21.5 ± 1.9	25 ± 3.2	20.1 ± 2.1	24 ± 3.7
AST	123.7 ± 16.4	175 ± 31.6	269 ± 21.4^∗^	285 ± 25.4^∗^	228 ± 21.6^∗^
ALT	23.6 ± 2.1	22.8 ± 1.8	28.3 ± 1.8^∗^	29.2 ± 2.9^∗^	28.5 ± 2.4^∗^

Blood samples were taken after euthanasia (day 61). C1: control, water via gavage and standard chow diet; C2: control, water (no gavage) and standard chow diet; AL: alcohol (40%) via gavage and standard chow diet; HF: water (no gavage) and high-fat diet (50%); HF + AL: alcohol (40%) via gavage and high-fat diet. ∗ indicates statistical differences versus C1 and C2; # indicates statistical difference versus C1, C2, AL, and HF.

## Data Availability

The data used to support the findings of this study are available from the corresponding author upon request.
